# Year-Long Antibody Response to the EuCorVac-19 SARS-CoV-2 Vaccine in Healthy Filipinos

**DOI:** 10.3390/vaccines13080776

**Published:** 2025-07-22

**Authors:** Jonathan F. Lovell, Kazutoyo Miura, Yeong Ok Baik, Chankyu Lee, YoungJin Choi, Jeong-Yoon Lee, Carole A. Long, Michelle Ylade, Roxas Lee-Llacer, Norman De Asis, Mitzi Trinidad-Aseron, Jose Manuel Ranola, Loreta Zoleta De Jesus, Howard Her

**Affiliations:** 1 Department of Biomedical Engineering, University at Buffalo, Buffalo, NY 14260, USA; 2 Laboratory of Malaria and Vector Research, National Institute of Allergy and Infectious Diseases, National Institutes of Health, Rockville, MD 20892, USA; kmiura@niaid.nih.gov (K.M.); clong@niaid.nih.gov (C.A.L.); 3 EuBiologics, R&D Center, EuBiologics Co., Ltd., Seoul 06778, Republic of Korea; yobaek@eubiologics.com (Y.O.B.); cklee@eubiologics.com (C.L.); yjchoi@eubiologics.com (Y.C.); jyl880111@eubiologics.com (J.-Y.L.); hher@eubiologics.com (H.H.); 4 National Institutes of Health, University of the Philippines Manila, Manila 1000, Philippines; mcylade@up.edu.ph; 5 Bicol Regional Training and Teaching Hospital, Albay 4501, Philippines; raleellacer@gmail.com; 6 Norzel Medical & Diagnostic Clinic, Cebu City 6000, Philippines; normandeasis_md@yahoo.com; 7 University of Perpetual Help System DALTA, Manila 1740, Philippines; mitzi3nidad@gmail.com; 8 St John Hospital Inc., Naga 4400, Philippines; joeyranola@gmail.com; 9 Silang Specialists Medical Center, Cavite 4118, Philippines; balotlbz@yahoo.com

**Keywords:** SARS-CoV-2, vaccines, recombinant vaccines, liposomes, clinical trial

## Abstract

**Background:** We previously reported an interim safety and immunogenicity analysis of a Phase 3 trial in the Philippines of the EuCorVac-19 (ECV-19) COVID-19 vaccine with the COVISHIELD^TM^ (CS) comparator (ClinicalTrials.gov identifier NCT05572879). Here, we present full-year humoral immunogenicity analysis. **Methods**: Healthy adults over 18 years of age received two injections of ECV-19 or CS vaccines, with 4 weeks between prime and boost. Analysis was carried out in individuals with immunogenicity measurements available at all 4 timepoints (weeks 0, 6, 30, and 56; *n* = 535 for ECV-19 and *n* = 260 for CS). **Results**: 2 weeks after boosting (week 6), ECV-19 elicited higher median anti-RBD IgG (1512 vs. 340 BAU/mL, *p* < 0.001) and neutralizing antibodies (1280 vs. 453 median microneutralization (MN) titer, *p* < 0.001) compared to CS. Anti-RBD IgG remained higher for ECV-19 compared to CS through week 30 (412 vs. 238 BAU/mL, *p* < 0.001) and 56 (425 vs. 260 BAU/mL, *p* < 0.001). MN titers remained higher for ECV-19 compared to CS through week 30 (640 vs. 453, *p* < 0.001) and 56 (453 vs. 320, *p* < 0.001). Correlation between anti-RBD IgG and neutralization titers persisted throughout the study. Women generally exhibited greater antibody responses than men. In the first six months following immunization, the ECV-19 group had a median antibody half-life of 80 days for anti-RBD IgG and 112 days for MN titer. In the subsequent six months, antibody half-life increased to 237 days for anti-RBD IgG and 168 days for MN titer. **Conclusions**: Following initial prime-boost vaccination, ECV-19 maintained higher anti-RBD IgG and neutralizing antibody titers relative to the CS comparator over a full-year period.

## 1. Introduction

The COVID-19 pandemic has underscored the critical need for safe and effective vaccines capable of inducing long-lasting immunity [1]. While several vaccine platforms have demonstrated robust short-term responses against SARS-CoV-2 [2], data on their sustained immunogenicity over extended periods in diverse populations remains somewhat limited. Several longitudinal COVID-19 vaccine studies have focused on the impact of multiple boosting regimes [3,4,5], as opposed to assessing the long-term (e.g., year-long) durability of initial immunization. Prior studies in the Philippines have shown elicitation of antibodies in response to COVID-19 infection [6], to vaccines where participants received CoronaVac (Sinovac) and Comirnaty (Pfizer) [7] and shown indirect vaccine efficacy of SCB-2019 (Clover Biopharmaceuticals) [8].

Vaccines have proven to be powerful tools in controlling COVID-19, with various platforms, including mRNA vaccines, viral vectors, and protein subunits, demonstrating efficacy [9]. Protein subunit vaccines such as EuCorVac-19 (ECV-19) offer positive characteristics, including putatively favorable safety profiles, and simplified cold chain requirements. ECV-19 employs lipid bilayer display technology to present the receptor-binding domain (RBD) of the SARS-CoV-2 Spike protein on the surface of immunogenic liposomes [10,11]. The display is based on chelation of cobalt within an incorporated porphyrin-phospholipid molecule that coordinates with the polyhistidine tag of the protein antigen [12]. Antibodies against the RBD, generally correlate well with neutralizing activity, making it a target for vaccine design. By focusing on the RBD, ECV-19 aims to elicit a robust and durable immune response tailored to neutralize the virus effectively. Understanding the long-term (e.g., 1 year) durability of immune responses elicited by ECV-19 could be beneficial for informing vaccine deployment strategies, particularly in low- and middle-income countries.

Interim Phase 3 analyses in our previous study highlighted the superior immunogenicity of ECV-19 in comparison to COVISHIELD^TM^ (CS) at an early time point following primary vaccination [13]. CS is based on modified chimpanzee adenoviral vector-based vaccine technology [14]. The prior interim findings demonstrated higher binding antibody titers and higher neutralizing antibody response, positioning ECV-19 as a promising vaccine candidate. Following these results, this current study reports on the long-term immunogenicity of ECV-19 over a full year. The methodology used in this study to assess year-long antibody responses has not yet been applied to a larger Phase 3 trial, and may be useful for assessing the durability of COVID-19 vaccines. Our findings reveal that the superior immunogenicity observed with ECV-19 relative to CS at interim stages was maintained throughout the one-year follow-up period.

## 2. Materials and Methods

### 2.1. Study Design

Healthy male and female participants aged 18 years and older were eligible and were enrolled from September 2022, with the last biospecimen collection completed February 2024. Details of the trial design and methodology were previously reported [13]. In brief, this study was conducted as a Phase 3, randomized, observer-blind, active-controlled, parallel-group, multi-center study at six clinical sites across the Philippines (ClinicalTrials.gov identifier NCT05572879). The trial adhered to the ethical guidelines of Good Clinical Practice as outlined in the International Council for Harmonisation (ICH) E6(R2) Integrated Addendum. Approvals were obtained from the Philippine Food and Drug Administration and the Institutional Review Boards (IRBs) of the participating clinical sites. The trial registration can be found on ClinicalTrials.gov (NCT05572879).

At the initial visit (V1), participants provided written informed consent. Baseline data, including medical history, vital signs, height, weight, and laboratory results (hematology, blood chemistry, urinalysis, blood coagulation), were collected, along with a pregnancy test for women of childbearing potential. Participants meeting eligibility criteria were assigned to either the safety or immunogenicity cohort, based on the randomization method previously described [13]. Immunogenicity analysis was only performed in the immunogenicity cohort. Participants then received their first dose of either CS (ChAdOX1 nCoV-19 recombinant coronavirus vaccine, 5 × 10^10^ viral particles) or ECV-19 (20 µg recombinant RBD protein with 40 µg cobalt-porphyrin phospholipid (CoPoP) and 20 µg *E. coli* produced monophosphoryl lipid A (EcML)) at the second visit (V2; week 0). Blood samples were collected at weeks 0, 4 (second dose), 6 (two weeks after the second dose), 30 and 56. However, week 4 data has been published previously [13], and was not used in the current analysis. Immediate adverse events (AEs) were monitored for at least 30 min (2 h for participants aged 75 years or older) post-vaccination, while solicited and unsolicited AEs were recorded through participant diary cards and follow-up visits up to 4 weeks after the 2nd vaccination, and SAEs, MAAEs, and AESIs were recorded and followed for up to week 56 from the 1st vaccination. AE severity was graded using the Ministry of Food and Drug Safety guidelines for vaccine trials, and all AE occurred by week 6 have been reported previously [13].

Serum samples were sent to VisMederi (Siena, Italy) for analysis under Good Clinical Laboratory Practice (GCLP) and ISO 9001:2015 standards [13]. RBD-specific ELISA titers were measured using a microplate-based colorimetric assay, with homologous antigen coating and signal detection processes conducted according to standard protocols. Titers were converted to RBD BAU/mL using the WHO International Standard (NIBSC code 20/136). Neutralizing antibody analysis was performed using a cytopathic effect (CPE) microneutralization assay with ancestral SARS-CoV-2 and Omicron BA.5 variants, with results reported as microneutralization (MN) titers.

### 2.2. Statistical Analyses

Statistical analyses were conducted using SAS software (version 9.4 or higher) and GraphPad Prism (version 9). Differences between groups were analyzed with a two-proportion z-test or Mann–Whitney test, and within-group comparisons over time employed Friedman tests followed by Dunn’s post hoc analyses. Correlations were examined with Spearman rank tests, while group proportions were analyzed with Fisher’s exact test. A *p*-value of less than 0.05 was considered statistically significant.

## 3. Results

The interim immunogenicity and safety findings were reported previously [13]. In the full year study, the status of serious adverse events (SAEs), Medically Attended Adverse Events (MAAEs), and Adverse Events of Special Interests (AESIs) was assessed up to 1 year after the second administration of study vaccines. Safety analysis showed that the ECV-19 and CS groups had comparable safety profiles. There was a total of 27 SAEs in 14 participants during the study, including 18 SAEs in participants (10/2004, 0.5%) of the ECV-19 group and 9 SAEs in participants (4/596, 0.7%) of the CS group. There were three deaths in the ECV-19 group and one in the CS group. All SAEs and deaths were determined to be not related to the study vaccines. See Supporting Table S1 for a list of deaths and Supporting Table S2 for a summary of SAEs.

In the previous interim report, the immunological results up to week 6 were analyzed using data from *n* = 585 (ECV-19 group) and *n* = 290 (CS group) individuals (interim report (IR) set). In this study, data from individuals for whom immunological measurements were available at all four timepoints (weeks 0, 6, 30, and 56) were utilized (full year (FY) set); *n* = 530 for ECV-19 group (91% of IP set) and *n* = 258 for CS group (89%). Participant demographics and immunological measurements at week 6 in both data sets are shown in Table 1. Nearly the entire study population (98%) had previously been exposed to SARS-CoV-2 prior to receiving the first immunization, as described in the interim study [13]. There was no significant difference between the IR set and FY set for the sex ratio, age, anti-RBD titers, and microneutralization (MN) titers against Wuhan strain (Wu-MN titers), suggesting there was no selection bias in this study.

**Figure 1:**
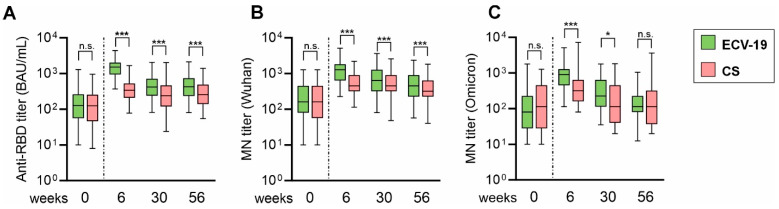
Anti-RBD and microneutralization titers remained higher in ECV-19 group relative to the CS. ECV-19 and CS vaccines were given at weeks 0 and 4, and serum samples were collected from ECV-19 (*n* = 530) and CS (*n* = 258) groups at weeks 0, 6, 30, and 56. IgG antibody levels against the RBD were measured by ELISA (**A**), and neutralization activity against the Wuhan strain (**B**) and the Omicron strain (**C**) was compared using microneutralization (MN) titers. Only a subset of samples (*n* = 63 for ECV-19, and *n* = 26 for CS) was tested against the Omicron strain. Box plots (25/50/75 percentiles) and 2.5/97.5 percentiles (error bars) are shown. The difference between ECV-19 and CS at each time point was assessed by a Mann–Whitney test. *** *p* < 0.001; * *p* < 0.05; n.s., not significant.

As expected, anti-RBD titers and Wu-MN titers decreased over time both in ECV-19 and CS groups overall (Figure 1). When data from the two groups were compared at each time point, the ECV-19 group maintained significantly higher anti-RBD titers at weeks 30 and 56 compared to the CS group. The median titers in ECV-19 group were 418 and 426 BAU/mL at weeks 30 and 56, respectively, while those in CS group were 238 and 258, respectively. A similar trend was seen in Wu-MN titers; median MN titers in ECV-19 groups were 640 (week 30) and 453 (week 56), and those in CS group were 453 (week 30) and 320 (week 56). The ECV-19 group maintained significantly higher neutralizing antibodies compared to the CS group at weeks 30 and 56. A small subset of sera was tested against the Omicron strain (*n* = 63 for ECV-19 and *n* = 26 for CS group), and demonstrated a significant difference at week 30 (median titers of 226 vs. 113 for ECV-19 and CS groups, respectively), while there was no difference at week 56 (113 median titers for both groups).

When anti-RBD titers at weeks 30 and 56 were compared with those at week 0 (pre-vaccination) within a group, the week 30 and 56 titers remained significantly higher than week 0 titers both for ECV-19 and CS groups (Supporting Table S3). Similarly, Wu-MN titers were significantly higher at weeks 30 and 56 compared to those at week 0 for both groups. On the other hand, MN titers against the Omicron strain (Om-MN titers) went back to the pre-vaccination levels at week 30 in CS group (*p* > 0.999), and at week 56 (*p* > 0.999) in ECV-19 group. In the ECV-19 group, the week 30 Om-MN titer was significantly higher than week 0 titer, *p* < 0.001.

**Figure 2:**
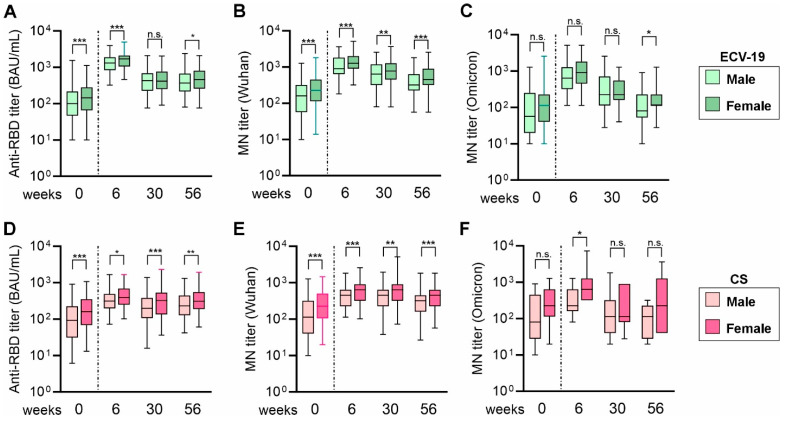
Effect of sex on humoral immune responses. The data presented in Figure 1 were divided by sex. For anti-RBD titers and MN titers against Wuhan, data from 252 males and 278 females were analyzed for ECV-19 group (**A**,**B**), and those from 152 males and 106 females for CS group (**D**,**E**). For MN titers against Omicron, data from 30 males and 33 females were analyzed for ECV-19 group (**C**), and those from 19 males and 7 females for CS group (**F**). Box plots (25/50/75 percentiles) and 2.5/97.5 percentiles (error bars) are shown. The difference between males and females at each time point was assessed by a Mann–Whitney test. *** *p* < 0.001; ** *p* < 0.01; * *p* < 0.05; n.s., not significant.

A significant effect of sex on immunological measurements (i.e., females showed significantly higher titers than men) was seen in the early time points (weeks 0, 4, and 6) of this phase 3 trial [13] and all time points (up to 1 year) in the previous phase 2 trial [15]. Therefore, male and female data at weeks 30 and 56 were also compared in this study (Figure 2). Similarly to the abovementioned studies, anti-RBD IgG titers were significantly higher in females at both time points for CS group. On the other hand, in ECV-19 group, while the same phenomenon was seen at week 56, there were no differences between males and females at week 30. For the Wu-MN titers, the differences were significant for both vaccine groups at both time points. For the Om-MN titers, the median titers in females were higher than those in males at weeks 0, 6, and 56 for both groups. However, since many fewer samples were tested against the Omicron strain, the differences did not reach significance, except for week 56 in ECV-19 group and week 6 in CS group.

**Figure 3:**
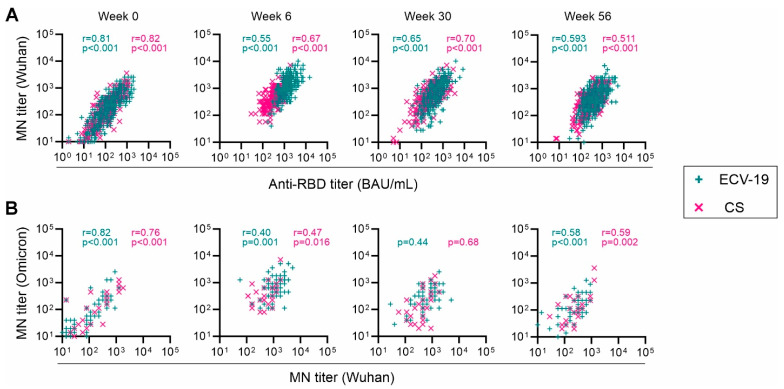
Immunological correlations between anti-RBD titer and neutralizing titers throughout the study. A correlation between anti-RBD titer and MN titers (Wuhan) (**A**) and MN titers against two strains (**B**) at each time point was examined by Spearman’s rank test. When the correlation is significant, Spearman’s rank correlation coefficient (r) is also shown.

Next, we evaluated associations among the immunological readouts within this study. Consistent with our previous observations [11], there were strong correlations between anti-RBD titers and Wu-MN titers throughout the study for both ECV-19 and CS groups (*p* < 0.001 and Spearman’s rank correlation coefficients, r, were >0.5 for both groups; Figure 3A). Significant correlations between Wu-MN titers and Om-MN titers were also observed, except for Week 30 (Figure 3B).

Vaccine-elicited antibody durability can be assessed by determining antibody half-life, the period in which antibody levels decay to half the initial concentration. To determine the half-life of anti-RBD titers and Wu-MN titers in this population, the changes from week 6 to 30 sampling time points (called “early decay period”), and those from week 30 to 56 sampling timepoints (“late decay period”) were investigated individually (Figure S1). During the early decay period, a significantly smaller proportion of people in CS group demonstrated decreases in titers. In ECV-19 group, 94% of individuals showed a decrease in anti-RBD titers, while 69% in CS group (*p* < 0.0001 by Fisher’s exact test). For anti-Wu-MN titers, 68% in ECV-19 group and 43% in CS group demonstrated a decrease in titers (*p* < 0.0001). On the other hand, during the late decay period, similar proportions of individuals in both groups showed a decrease in anti-RBD titers (51% in ECV-19 group and 43% in CS group, *p* = 0.049) or anti-Wu-MN titers (63% in ECV-19 and 63% in CS groups, *p* = 0.937).

**Figure 4:**
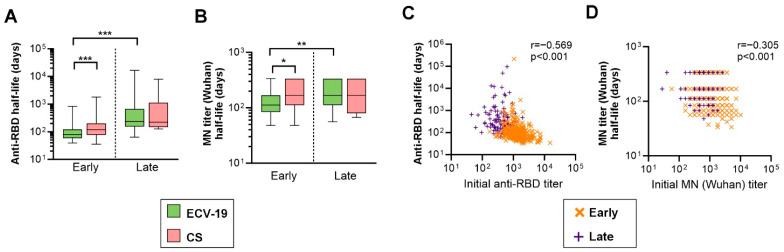
Antibody half-life during early and late decay period. For individuals who showed decrease in titers over time, half-life of anti-RBD titer (**A**) and anti-MN (Wuhan) titer (**B**) were calculated during 6–30 weeks (early) or during 30–56 weeks (late) decay period using an exponential decay model. The numbers of individuals used for the analysis were 352 (ECV-19 early), 100 (ECV-19 late), 115 (CS early), and 10 (CS late). A significant difference was examined by a Kruskal–Wallis test followed by Dunn’s multiple comparisons test.*** *p* < 0.001; ** *p* < 0.01; * *p* < 0.05. (**C**,**D**) The correlation between initial titer (titers at week 6 for early decay half-life data, and those at week 30 for late decay half-life data) and the corresponding half-life was evaluated for ECV-19 group (a total of 467 data points in each panel). Spearman’s rank *p*-value and correlation coefficient (r) are shown in each panel by combining early and late decay data. The same plots for CS group are shown in Figure S2.

While COVID-19 infection and vaccination history were not collected after week 6 in this study, no reduction in either of titers during the 24 (early decay period) or 26 (late decay period) weeks suggests that they were exposed to natural infection(s) and/or received another COVID-19 vaccination(s) during the period. Therefore, using the same methodology developed in our previous analysis of the durability of ECV-19 in the Phase 2 trial [15], such individuals were excluded from the half-life analysis. As a result, the half-life of the early decay period was calculated for 352 (66% of 530) individuals in ECV-19 group and 100 (39% of 258) individuals in CS group using a one-phase exponential decay model (Figure 4A,B). There was a significant difference in anti-RBD half-life (*p* < 0.0001) between ECV-19 (median 80 days; Interquartile range, IQR, 59–123 days) and CS (120; 78–199 days) groups. There was a significant difference (*p* = 0.0127) in anti-Wu-MN half-life between ECV-19 (112; 84–168 days) and CS (168; 112–336 days) groups as well. For the half-life in the late decay period, further exclusion was conducted if individuals did not demonstrate a reduction in titers during the study period, ending up with 115 individuals for ECV-19 and 10 individuals for CS groups. Since there were only 10 data points for CS group, no statistical comparison was made to the data. Within the ECV-19 group, both anti-RBD half-life (237; 154–671 days) and anti-Wu-MN half-life (168; 112–336 days) were significantly longer (*p* < 0.003 for both) during the late decay period compared to those in early decay period as expected (Figure 4A,B). For ECV-19 group, as observed in the phase 2 trial [15], there was a significant negative correlation between initial titers (titers at week 6 for early decay half-life data, and those at week 30 for late decay half-life data) and corresponding half-life data (*p* < 0.001; Figure 4C,D). In other words, individuals with higher initial titers showed shorter antibody half-life. On the other hand, the correlation for anti-RBD titers in CS group barely did not reach significance (*p* = 0.071) and there was no correlation in anti-Wu-MN titers (*p* = 0.804) (Figure S2). The insignificant correlations seen in CS group might be explained by the lower titers overall and/or fewer data points for the CS group.

## 4. Discussion

ECV-19 robustly elicited anti-RBD IgG titers and MN titers that persisted over the subsequent 12-month period and were significantly greater than the CS comparator throughout. While both vaccines exhibited a decline in antibody levels over time, the titers for ECV-19 stabilized at significantly higher levels at 6 and 12 months post-boost compared to CS. This persistent elevation of anti-RBD IgG titers may point to advantages in terms of durability, particularly for populations requiring extended protection without frequent booster doses.

In this trial, nearly the entire study population (98%) had previously been exposed to SARS-CoV-2 and therefore started with a pre-existing anti-RBD antibody response prior to vaccination at week 0 (V2), as described in the interim study [13]. Potentially for this reason, we also observed no increase in anti-RBD antibody levels in CS or ECV-19 groups with the second vaccination that occurred at boosting 4 weeks after immunization [13]. It is therefore interesting that the higher antibody response observed for ECV-19 relative to CS during the interim period held up throughout an entire year.

The prior Phase 2 trial of ECV-19 was conducted in Korea as opposed to the Philippines, and in contrast to this study, no participants had prior SARS-CoV-2 exposure [15]. An early and late decay period neutralizing half-life of 120 and 214 days, respectively, was observed in that study. Those results are generally consistent with the current study, which showed an early and late period neutralizing antibody half-life of 112 and 168 days, respectively. An overall neutralizing antibody half-life of 90 days in the 8 months following natural infection has been suggested [16]. To the best of our knowledge, there are few reports of SARS-CoV-2 vaccine half-lives over a one-year follow-up period, potentially due to challenges associated with the impact of reinfection on data analysis, which our methodology addresses by removing any subjects with increasing antibody levels.

The observation that women maintained higher titers than men at the 12-month mark of the study period also aligns with our previous analysis of year-long ECV-19 immunogenicity in the Phase 2 trial [15]. We also observed that the CS comparator elicited higher persistent antibody responses in females compared to males. This viral vector vaccine was found to elicited slightly higher anti-Spike IgG immunogenicity in females in analysis of trials in Brazil and UK [17]. It has been reported that the COVID-19 mRNA vaccination elicits higher humoral responses in women compared to men [18]. In general, females typically develop higher antibody responses following vaccination than males [19].

This study has several limitations to mention. Notably, it did not assess clinical efficacy or the cellular immune response, which are crucial components of comprehensive vaccine evaluation. The lack of efficacy testing means the long-term protection offered by ECV-19 against SARS-CoV-2 infection or severe disease remains uncertain. Analyses have suggested that antibodies against the S1 domain of the Spike protein, which contains the RBD, offer the most accurate correlate of protection against infection by SARS-CoV-2 [20]. However, despite this correlation, using absolute measure of antibodies as a proxy for protective responses has not yet been established. Additionally, cellular responses, such as T-cell activation, were not evaluated, leaving an incomplete understanding of the vaccine’s immunogenic profile. Given the critical role of cellular immunity in durable protection, future studies should incorporate assays for CD4^+^ and CD8^+^ T-cell responses. Furthermore, the study’s generalizability may be limited due to the focus on a single geographic region, potentially overlooking variations in response among diverse populations. Addressing these gaps will be of benefit for fully characterizing ECV-19’s potential as a global immunization tool. Finally, the exclusion criteria for the half-life analysis (exclude data only if the titers stayed the same or increased during the analysis period) was imperfect, thus the true half-life values (when an individual did not have any exposure to the COVID-19 virus or vaccine) could be shorter than the reported values here. However, since the COVID-19 virus has become ubiquitous, it is practically very challenging to determine the true half-life.

While the commercial demand for COVID-19 vaccines is lower in 2025 than during the peak of the pandemic, there still is a need since the virus remain in wide circulation. There is the possibility that COVID-19 becomes an annual vaccination scheme similar to influenza immunization, with antigen updating depending on the circulating strain, however the policy for annual virus respiratory vaccines that includes SARS-CoV-2 remains to be established [21]. There has also been interest in co-administered or co-formulated vaccines for COVID-19, influenza virus and RSV [22]. ECV-19 currently makes use of the RBD derived from the Wuhan strain which is no longer in circulation. However, updating the RBD antigen to match the circulating strain could be adapted for future vaccine updates against variants. This 1-year follow-up on the interim Phase 3 trial results is important because it ultimately confirms the long-term immunogenicity and feasibility of this new type of liposome-displayed, adjuvanted recombinant subunit vaccine platform.

## 5. Conclusions

The ECV-19 vaccine elicited sustained and significantly higher anti-RBD IgG titers and neutralizing antibody titers compared to the CS viral vector comparator over a 12-month period, highlighting its potential for long-term humoral immunity. While these findings are promising, future studies are necessary to assess the efficacy and cellular responses to better gaugethe vaccine’s protective capabilities. Future studies addressing these gaps will be critical to understanding and optimizing ECV-19’s role in SARS-CoV-2 prevention strategies.

## Figures and Tables

**Table 1:** Participant demographics and week 6 immunological measurements.

	ECV-19			CS		
	IR Set ^a^	FY Set ^b^	*p*-Value ^c^	IR Set ^a^	FY Set ^b^	*p*-Value ^c^
Sex; Male/Female	275/310	252/278	0.90	169/121	152/106	0.88
Age; Median (range)	30 (18–69)	31 (18–69)	0.85	31 (18–66)	31 (18–66)	0.91
Anti-RBD titer; Median (IQR ^d^)	1509 (942–2017)	1512 (936–2024)	0.87	340 (214–525)	339 (210–525)	0.94
MN titer (Wuhan) ^e^; Median (IQR ^d^)	1280 (640–1810)	1280 (640–1810)	0.74	453 (320–905)	453 (320–905)	0.86

^a^ Data set used for previous interim report. ^b^ Data set used in this full-year study. ^c^ The *p*-value was calculated by Fisher’s exact test for Sex data, and by a Mann–Whitney test for the other data. ^d^ Interquartile range. ^e^ Microneutralization titers against Wuhan strain.

## Data Availability

The original contributions presented in this study are included in the article/Supplementary Materials. Further inquiries can be directed to the corresponding author.

## Supplementary Materials

The following supporting information can be downloaded at: https://www.mdpi.com/article/10.3390/vaccines13080776/s1, Figure S1: Reverse cumulative distribution plot for changes in anti-RBD and anti-MN (Wuhan) titers over time; Figure S2: Antibody half-lives during early and late decay period in CS group; Table S1: Summary of deaths; Table S2: Summary of Serious Adverse Events by System Organ Class; Table S3: Time-course analysis within a group.

## Abbreviations

The following abbreviations are used in this manuscript:

AE	Adverse Event
AESI	Adverse Event of Special Interest
BAU/mL	Binding Antibody Units per milliliter
CPE	Cytopathic Effect
CS	COVISHIELDTM
CoPoP	Cobalt-Porphyrin Phospholipid
COVID-19	Coronavirus Disease 2019
EcML	*E. coli*-produced Monophosphoryl Lipid A
ECV-19	EuCorVac-19
ELISA	Enzyme-Linked Immunosorbent Assay
FY	Full Year
GCLP	Good Clinical Laboratory Practice
ICH	International Council for Harmonisation
IR	Interim Report
IRB	Institutional Review Board
ISO	International Organization for Standardization
IP	Immunogenicity Population (inferred)
MAAE	Medically Attended Adverse Event
MN	Microneutralization
NIBSC	National Institute for Biological Standards and Control
RBD	Receptor-Binding Domain
SAE	Serious Adverse Event
SARS-CoV-2	Severe Acute Respiratory Syndrome Coronavirus 2
V1/V2	Visit 1/Visit 2
WHO	World Health Organization
Wu-MN	Wuhan Microneutralization
