# Supplementary material for: Year-Long Antibody Response to the EuCorVac-19 SARS-CoV-2 Vaccine in Healthy Filipinos
Source: Vaccines (Basel). 2025 Jul 22;13(8):776. doi: 10.3390/vaccines13080776 (PMC12389930; doi:10.3390/vaccines13080776)
Supplement: Supplementary file 1 [file vaccines-13-00776-s001.zip › vaccines-3739784-supplementary.pdf]

Supporting Information for: Year-long antibody response to the EuCorVac-19 SARS-CoV-2 vaccine in healthy Filipinos

Jonathan F. Lovell, Kazutoyo Miura, Yeong Ok Baik, Chankyu Lee, YoungJin Choi, Jeong-Yoon Lee, Carole A. Long, Michelle Ylade, Roxas Lee-Llacer, Norman De Asis, Mitzi Trinidad-Aseron, Jose Manuel Ranola, Loreta Zoleta De Jesus, Howard Her

Table S1: Summary of deaths

Treatment	Sex	Age (at Screening)	1st Vaccine Date	2nd Vaccine Date	Day / Date of death	Medical or Surgical History	Solicited AEs (Grade)	Primary Cause of Death	Determined Relationship of IP to Death
ECV-19	Male	30 years	Day 0 09 Nov 2022	Not administered	Day 19 29 Nov 2022	None	None	Acute coronary syndrome	Unrelated
ECV-19	Male	28 years	Day 0 25 Oct 2022	Day 28 23 Nov 2022	Day 126 01 Mar 2023	None	Headache (Grade 2) on Day 4 Headache (Grade 1) on Day 5	Acute coronary syndrome	Unrelated
ECV-19	Male	36 years	Day 0 11 Nov 2022	Day 28 09 Dec 2022	Day 391 08 Dec 2023	None	None	Road traffic accident	Unrelated
CS	Male	63 years	Day 0 06 Dec 2022	Day 28 03 Jan 2023	Day 366 06 Dec 2023	Hypertensive heart disease	None	Status asthmaticus	Unrelated

Table S2: Summary of Serious Adverse Events by System Organ Class

**System Organ Class / Preferred Term**	**ECV-19 Total (N=2004) n (%) m**	**COVISHIELD Total (N=596) n (%) m**
Participants with at least 1 SAE	10 (0.5) 18	4 (0.7) 9
Gastrointestinal Disorders	3 (0.1) 3	1 (0.2) 2
Upper gastrointestinal haemorrhage	1 (0.1) 1	0
Incarcerated inguinal hernia	0	0
Small intestinal obstruction	0	0
Stress ulcer	0	1 (0.2) 1
Blood and lymphatic system disorders	2 (0.1) 2	1 (0.2) 1
Anaemia	2 (0.1) 2	0
Pregnancy, puerperium and perinatal conditions	1 (0.1) 1	2 (0.3) 2
Abortion spontaneous	0	2 (0.3) 2
Foetal death	2 (0.1) 2	0
Vascular disorders	2 (0.1) 2	0
Hypertension	2 (0.1) 2	0
Cardiac disorders	2 (0.1) 2	0
Acute coronary syndrome	2 (0.1) 2	0
Infections and infestations	1 (0.1) 3	0
Pneumonia	1 (0.1) 1	0
Bone tuberculosis	1 (0.1) 1	0

Pulmonary tuberculosis	1 (0.1) 1	0
Injury, poisoning and procedural complications	1 (0.1) 1	0
Road traffic accident	1 (0.1) 1	0
Metabolism and nutrition disorders	0	0
Type 2 diabetes mellitus	0	0
Musculoskeletal and connective tissue disorders	0	0
Muscular weakness	0	0
Neoplasms benign, malignant and unspecified	1 (0.1) 1	0
Ovarian germ cell teratoma benign	1 (0.1) 1	0
Nervous system disorders	0	0
Cerebral infarction	1 (0.1) 1	0
Respiratory, thoracic and mediastinal disorders	0	0
Status asthmaticus	0	1 (0.2) 1
Skin and subcutaneous tissue disorders	1 (0.1) 1	0
Diabetic foot	1 (0.1) 1	0

Table S3. Time-course analysis within a group. Immunological data shown in Fig 1 were analyzed in each group at different time points by a Friedman test followed by Dunn's multiple comparisons test. The Friedman p-values were <0.001 for all data sets, and Dunn's multiple comparisons test results are shown. ***, p<0.001; **, p<0.01; *, p<0.05; n.s., not significant.

	ECV-19						CS					
Anti-RBD titer	*Weeks*	*0*	*6*	*30*	*56*	*median titer*	*Weeks*	*0*	*6*	*30*	*56*	*median titer*
	0					126	*0*					125
	6	***				1,512	*6*	***				839
	30	***	***			418	*30*	***	****			238
	56	***	***	n.s.		426	*56*	***	**	n.s.		258
MN titer (Wuhan)	*Weeks*	*0*	*6*	*30*	*56*	*median titer*	*Weeks*	*0*	*6*	*30*	*56*	*median titer*
	0					160	*0*					160
	6	***				1,280	*6*	***				453
	30	***	***			640	*30*	***	n.s.			453
	56	***	***	***		453	*56*	***	***	***		320
MN titer (Omicron)	*Weeks*	*0*	*6*	*30*	*56*	*median titer*	*Weeks*	*0*	*6*	*30*	*56*	*median titer*
	0					80	*0*					113
	6	***				905	*6*	*				320
	30	***	***			226	*30*	n.s.	n.s.			113
	56	n.s.	***	***		113	*56*	n.s.	***	n.s.		113

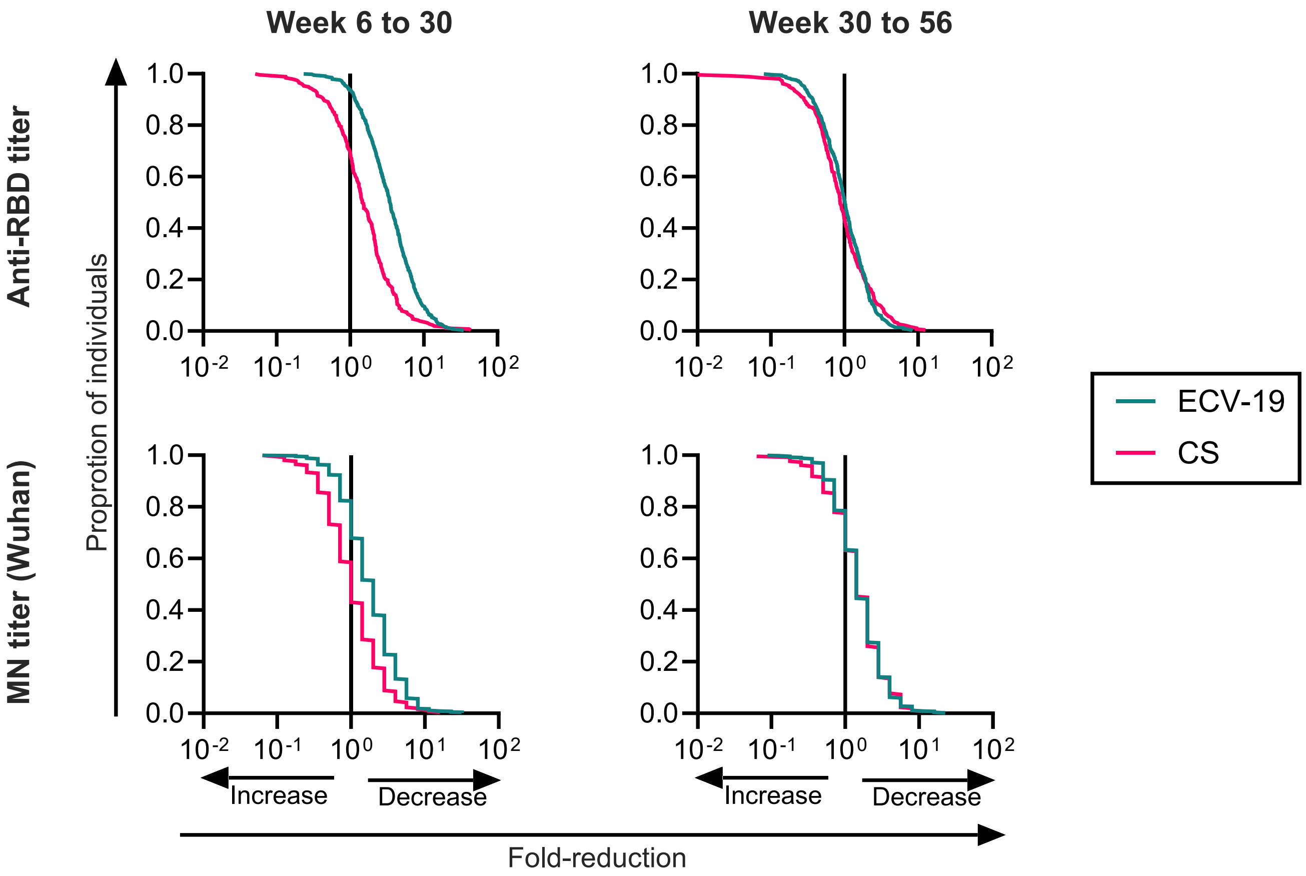

Figure S1. Reverse cumulative distribution plot for changes in anti-RBD and anti-MN (Wuhan) titers over time. In each individual, a change in anti-RBD or anti-MN titers from week 6 to 30 and those from week 30 to 56 were calculated. The results were presented in a reverse cumulative distribution plot. For example, the top left panel demonstrates that 94% of individuals showed a decrease in anti-RBD titers from weeks 6 to 30 in ECV-19 group, while 68% showed a decrease in the titers in CS group during the same period.

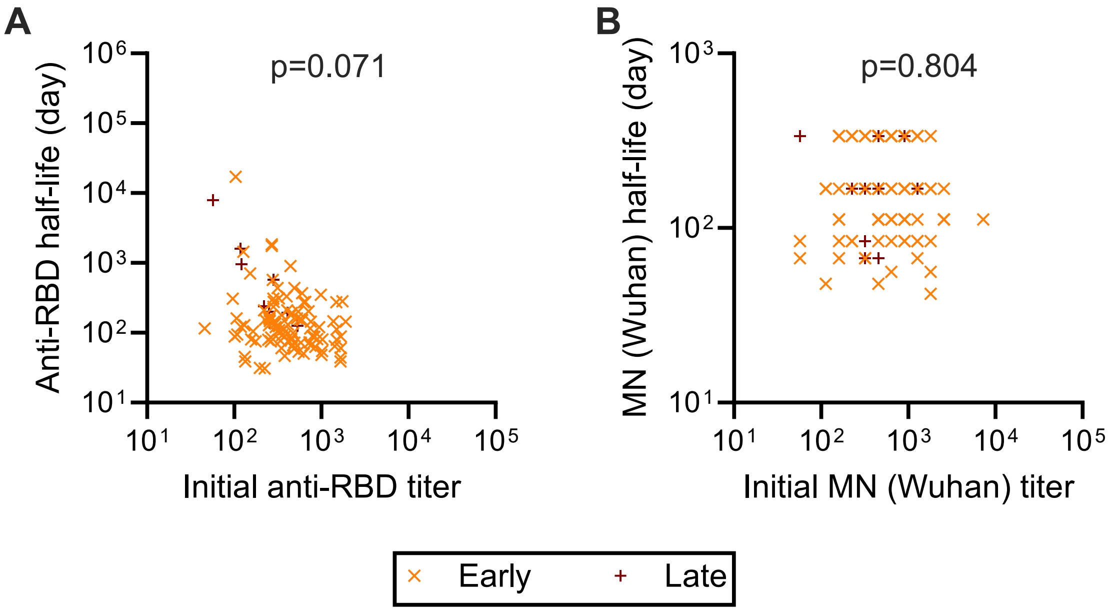

Figure S2. Antibody half-lives during early and late decay period in CS group. Similar plots as Fig 4C and 4D, but represent data for CS group (a total of 110 data points in each panel). A Spearman rank p-value is shown in each panel by combining early and late decay data.
